# Laminin-guided highly efficient endothelial commitment from human pluripotent stem cells

**DOI:** 10.1038/srep35680

**Published:** 2016-11-02

**Authors:** Ryo Ohta, Akira Niwa, Yukimasa Taniguchi, Naoya M. Suzuki, Junko Toga, Emiko Yagi, Norikazu Saiki, Yoko Nishinaka-Arai, Chihiro Okada, Akira Watanabe, Tatsutoshi Nakahata, Kiyotoshi Sekiguchi, Megumu K. Saito

**Affiliations:** 1 Department of Clinical Application, Center for iPS Cell Research and Application, Kyoto University, Kyoto, Japan; 2 Laboratory of Extracellular Matrix Biochemistry, Institute for Protein Research, Osaka University, Suita, Japan; 3 Depeartment of Life Science Frontiers, Center for iPS Cell Research and Application, Kyoto University, Kyoto, Japan; 4 Mitsubishi Space Software CO., LTD, Amagasaki, Japan

## Abstract

Obtaining highly purified differentiated cells via directed differentiation from human pluripotent stem cells (hPSCs) is an essential step for their clinical application. Among the various conditions that should be optimized, the precise role and contribution of the extracellular matrix (ECM) during differentiation are relatively unclear. Here, using a short fragment of laminin 411 (LM411-E8), an ECM predominantly expressed in the vascular endothelial basement membrane, we demonstrate that the directed switching of defined ECMs robustly yields highly-purified (>95%) endothelial progenitor cells (PSC-EPCs) without cell sorting from hPSCs in an integrin-laminin axis-dependent manner. Single-cell RNA-seq analysis revealed that LM411-E8 resolved intercellular transcriptional heterogeneity and escorted the progenitor cells to the appropriate differentiation pathway. The PSC-EPCs gave rise to functional endothelial cells both *in vivo* and *in vitro*. We therefore propose that sequential switching of defined matrices is an important concept for guiding cells towards desired fate.

The ECM forms the basal membrane to which it anchor cells *in vivo*, thereby regulating cellular survival, proliferation and differentiation by transducing various signals via cell surface receptors^1^. Because of these properties, various ECM coatings have been used for both embryonic tissue- and PSC-derived differentiation cultures. However, the selection and combination of ECMs for *in vitro* culture are determined rather empirically compared with other parameters, such as the cytokine levels and medium, yet must be well defined for directed differentiation from PSCs. Although optimizing the ECM coating at each *in vitro* stage is considered important for directed differentiation, there is little understanding of how the selection and switching of culture matrices determines the fate of progenitor cells.

Vascular endothelial cells (ECs) differentiated from PSCs have potential benefits for regenerative medicine of vascular diseases as well as *in vitro* disease modeling with patient-derived induced pluripotent stem cells (iPSCs), and a number of protocols for deriving ECs have been developed^2^,^3^,^4^. In the present work, we show the optimization of orderly endothelial cell development could be achieved by switching matrices during differentiation.

## Result

### Successful endothelial cell induction in conventional 2D method

Since a monolayer and feeder-free differentiation system suitable for exploring the role and effect of coating matrices, we first applied our feeder- and serum-free monolayer hematopoietic cell differentiation system on Matrigel^5^,^6^ for selective endothelial differentiation. This system develops VE-cadherin^+^ ECs concomitantly with hematopoietic cells from mesodermal progenitors^5^ (Supplementary Fig. 1a). Indeed, sequential cytokine switching successfully produced KDR^+^CD34^+^VE-cadherin^+^ PSC-EPCs (Supplementary Fig. 1b). Subsequent culture induced functional PSC-derived ECs that expressed the endothelial marker CD31 and incorporated acetyl-low-density-lipoprotein (Ac-LDL) on day 10 (Supplementary Fig. 1c), indicating successful differentiation into functional ECs. However, the efficiency for inducing PSC-EPCs was very low (approximately 10%) despite successful initial commitment to the mesodermal lineage (>80% of cells were KDR+ on day 3, Supplementary Fig. 1d) and subsequent VEGF stimulation.

### Discovery of coating condition appropriate for endothelial differentiation from mesodermal progenitors

**Figure 1:**
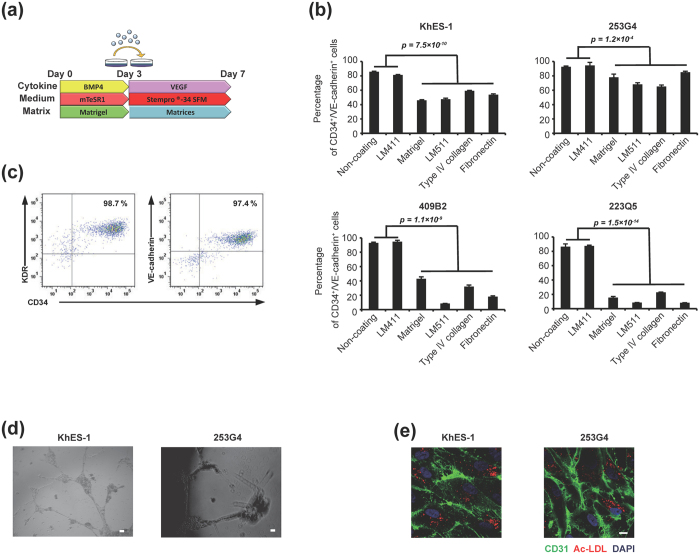
Differentiation of PSC-EPCs from human pluripotent stem cells using directed matrix switching. (**a**) Schematic outline of endothelial cell differentiation. (**b**) Purity of PSC-EPCs on day 7. Data are presented as the mean ± SEM (n = 3) and were statistically analyzed using ANOVA test. (**c**) Representative flow cytometry plots of cells (day 7) on LM411 (KhES-1). (**d**) Tube formation assay of PSC-ECs induced on LM411. Scale bar: 200 μm. (**e**) Ac-LDL-uptake and CD31 expression of PSC-ECs induced on LM411. Scale bar: 10 μm.

Since the vast majority of day 3 cells were positive for KDR, we next explored more appropriate conditions for their differentiation to endothelial lineage. We investigated various matrices onto which day 3 cells were plated and cultured for an additional 4 days in the presence of VEGF. As a result, we found that the non-coated and laminin 411 (LM411)-coated conditions reproducibly induced endothelial commitment with higher purity than other conditions (Fig. 1a–c, Supplementary Fig. 2). Of particular note, LM411 reproducibly presented a higher yield than the non-coated condition while maintaining comparable purity (Supplementary Fig. 3). The ECs derived from PSC-EPCs on LM411 possessed the capacities for Ac-LDL uptake and endothelial tube formation (Fig. 1d,e). Interestingly, matrices suitable for undifferentiated human PSCs such as Matrigel and laminin 511(LM511)^7^ showed relatively low purity (Fig. 1b), while LM411 could not support PSCs (data not shown). Taken together, these results demonstrated that LM411 acts as a suitable matrix for producing highly purified PSC-EPCs from mesodermal progenitors in day3 cells.

### The LM411-E8 fragment improved the endothelial cell yield and angiogenesis capacity

**Figure 2:**
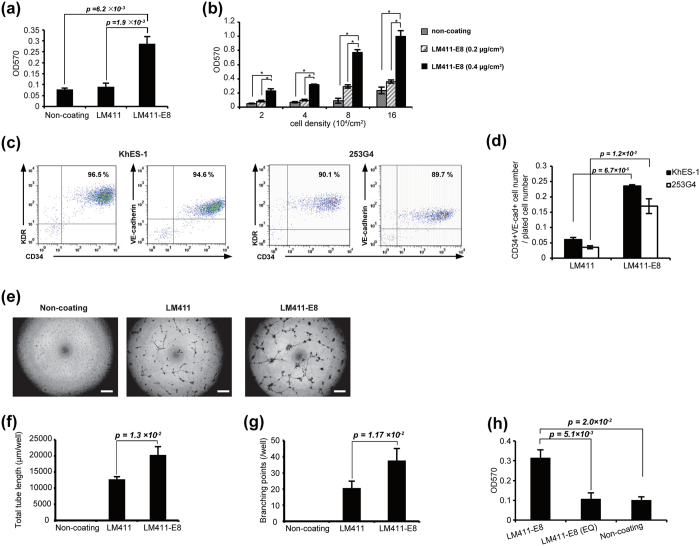
Functional properties of LM411-E8 for endothelial differentiation. (**a**) Cell adhesion assay of day 3 PSC-MPCs (KhES-1). Data are presented as the mean ± SEM (n = 3) and were statistically analyzed using Student’s *t*-test. Representative results of at least three independent experiments are shown. (**b**) Effect of coating density of LM411-E8 on the adhesion of PSC-MPCs derived from KhES-1. (**c**) Representative flow cytometry plots of cells (day 7) on LM411-E8. (**d**) Estimated yield of PSC-EPCs from one PSC-MPC. Data are presented as the mean ± SEM (n = 3) and were statistically analyzed using Student’s *t*-test. Representative results of at least three independent experiments are shown. (**e**–**g**) Evaluation of angiogenesis properties of PSC-ECs differentiated on non-coated dishes, LM411 or LM411-E8. (**e**) Representative images of tube-like structures formed by PSC-ECs derived from KhES-1. Scale bar: 500 μm. (**f**) Total tube length per well. Data are presented as the mean ± SEM (n = 3) and were statistically analyzed using Student’s *t*-test. Representative results of at least two independent experiments are shown. (**g**) Branching points per well. Data are presented as the mean ± SEM (n = 3) and were statistically analyzed using Student’s *t*-test. Representative results of at least two independent experiments are shown. (**h**) PSC-MPC adhesion was abrogated by LM411-E8 (EQ). Data are presented as the mean ± SEM (n = 3) and were statistically analyzed using Student’s *t*-test. Representative results of at least two independent experiments are shown.

Laminins are a common ECM component and responsible for various forms of cell-to-basement membrane adhesion^7^. There are 15 laminin isoforms in mammals including humans, among which laminin 411 (LM411) is the major isoform that lines the basal membrane of endothelial cells in capillary vessels and binds mainly to the cell surface transmembrane receptors integrin α6β1 and α7X1β1^8^. Based on the observation that laminins bind to integrins at their C-terminal region, we generated E8 fragments, which is the truncated form of the laminins that represent the C-terminal region^9^. E8 fragments retain full binding activity toward integrins but lack binding activities to other components, such as heparin/heparan sulfate. E8 fragments of LM511 and LM332 (LM511-E8 and LM332-E8, respectively) possess greater activity of PSC adhesion than their intact forms^10^. Accordingly, we next explored whether or not the LM411-E8 fragment (LM411E8) can improve the yield of PSC-EPCs (Supplementary Fig. 4a). Undifferentiated PSCs hardly adhered onto LM411-E8 coated dish, as with full-length LM411 (data not shown), but LM411-E8 showed significantly stronger adhesive properties with respect to day3 cells than did LM411 (Fig. 2a). In particular, cell adhesion increased in a dose-dependent manner, even with a higher cellular density (Fig. 2b), and the purity of PSC-EPCs on LM411-E8 continued to exceed 95%, which exceeded that on LM411, resulting in a significant increase in the cell number of PSC-EPCs (Fig. 2c,d, hereafter, mesodermal progenitors fitted to LM411-E8-mediated endothelial differentiation were referred to PSC-MPCs). Endothelial surface markers such as CD146^11^, CD143^12^, Tie-2^13^ and EphB4^14^ were expressed on day 9 cells, while a pluripotent marker Tra-1-60 was scarcely expressed (Supplementary Fig. 4b). Notably, PSC-EPCs derived on LM411-E8 yielded the endothelial cells that form longer and more branched tubes than those on LM411 (Fig. 2e–g, Supplementary Fig. 4c). In addition, PSC-EPCs on LM411-E8 also internalized Ac-LDL (Supplementary Fig. 4d).

In order to further evaluate the characteristics of PSC-EPCs on LM411-E8, we performed RNA-seq analysis of PSC-EPCs under several coating conditions. The transcriptional profiles of PSC-EPCs were closer to those of the primary endothelial cell-derived cell lines, such as HUVECs and HAECs, than parental ESCs (Supplementary Fig. 5a,b). As for specific gene expressions, some endothelial development-associated genes, such as LMO2, NOTCH1, HEY2, DLL4 and NRP1^11^,^15^,^16^, are upregulated, while function-associated genes, such as IL8, IL6, IL18, NFKB1, EDN1 and CAV1^17^,^18^,^19^, are downregulated in PSC-EPCs. However, representative common endothelial characteristic markers, such as CDH5, PECAM1 and MCAM, are expressed both on PSC-EPCs and primary ECs at comparable levels (Supplementary Fig. 5d). Moreover, the PSC-EPCs on LM411-E8 belonged to a cluster distinct from those on the full-length LM411/non-coating dish (Supplementary Fig. 5b), and a gene ontology analysis revealed that PSC-EPCs on LM411-E8 express lines of genes associated with vasculature development and angiogenesis at higher levels compared to those derived under other conditions (Supplementary Fig. 5c). Taken together, those data support the contribution of LM411-E8 to the endothelial induction in our system both in function, yield and purity.

### Initial adhesion of PSC-MPCs to LM411-E8 is dependent on integrin α6β1

We next explored the mechanisms underlying endothelial differentiation on LM411-E8. We first investigated whether the initial adhesion of PSC-MPCs onto LM411-E8 is dependent on the integrin-laminin axis, as adhesion to ligands other than integrins is usually abrogated in laminin-E8 fragments. For this purpose, we pre-treated PSC-MPCs with specific blocking antibodies against the α6 or β1 integrin subunits, both of which are expressed on PSC-MPCs (Supplementary Fig. 6a). Consequently, the adhered cells were significantly decreased to a level comparable to that observed under the non-coating condition (Supplementary Fig. 6b). Furthermore, we evaluated the degree of cell adhesion onto a mutant fragment of LM411-E8 (LM411-E8(EQ)), in which the Glu residue (E) in the C-terminal tail region of the γ1 chain of LM411-E8 was replaced with Gln (Q) to nullify integrin binding activity (Supplementary Fig. 6c). Notably, the adhesion of PSC-MPCs on LM411-E8 (EQ) was significantly decreased and comparable to that seen in the uncoated dishes (Fig. 2h). Overall, these data indicate that surface integrin family proteins, especially integrin α6β1, mediate the initial adhesion of PSC-MPCs onto LM411-E8.

### LM411-E8 guides PSC-MPCs into endothelial lineage in VEGF-dependent manner

**Figure 3:**
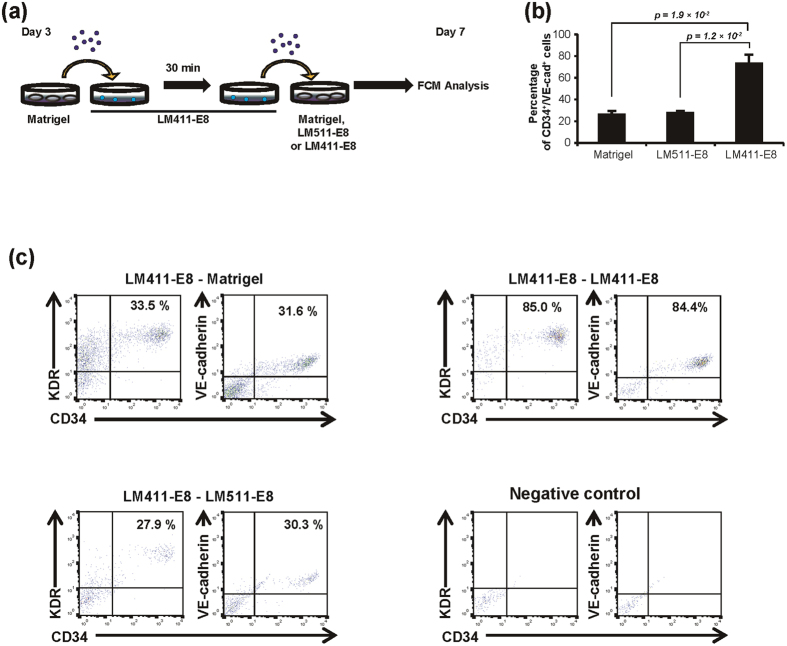
Effects of sequential switching of matrices during endothelial differentiation. (**a**) Schematic process of the double-switching assay. Day 3 cells were dissociated at the single-cell level and plated onto LM411-E8-coated plates. After 30 minutes of incubation at 37 °C, adherent cells were replated onto Matrigel, LM511-E8 and LM411-E8 and cultured with VEGF stimulation for four days. (**b**) Percentage of PSC-EPCs (KhES-1) differentiated from LM411-E8-selected mesodermal progenitors on Matrigel, LM511-E8 or LM411-E8 for four days. Data are presented as the mean ± SEM (n = 3) and were statistically analyzed using Student’s *t*-test. Representative results of at least two independent experiments are shown.

We next investigated whether LM411-E8 possess the ability to facilitate the endothelial differentiation of adhered PSC-MPCs. For this purpose, we carried out a double-switching assay (Fig. 3a) in which PSC-MPCs once adhered onto LM411-E8 were harvested again and subsequently seeded onto Matrigel, LM511-E8 or LM411-E8. LM411-E8 increased the purity of PSC-EPCs compared to Matrigel or LM511-E8 (Fig. 3b,c), indicating that LM411-E8 not only selects cells via integrin-dependent adhesion but also facilitates the endothelial differentiation of adhered progenitor cells. In the absence of VEGF, however, few PSC-EPCs were induced, even under the LM411-E8-coated condition (Supplementary Fig. 7).

### Single-cell RNA-seq analysis reveals involvement of Rho pathway in the endothelial differentiation of cells on LM411-E8

**Figure 4:**
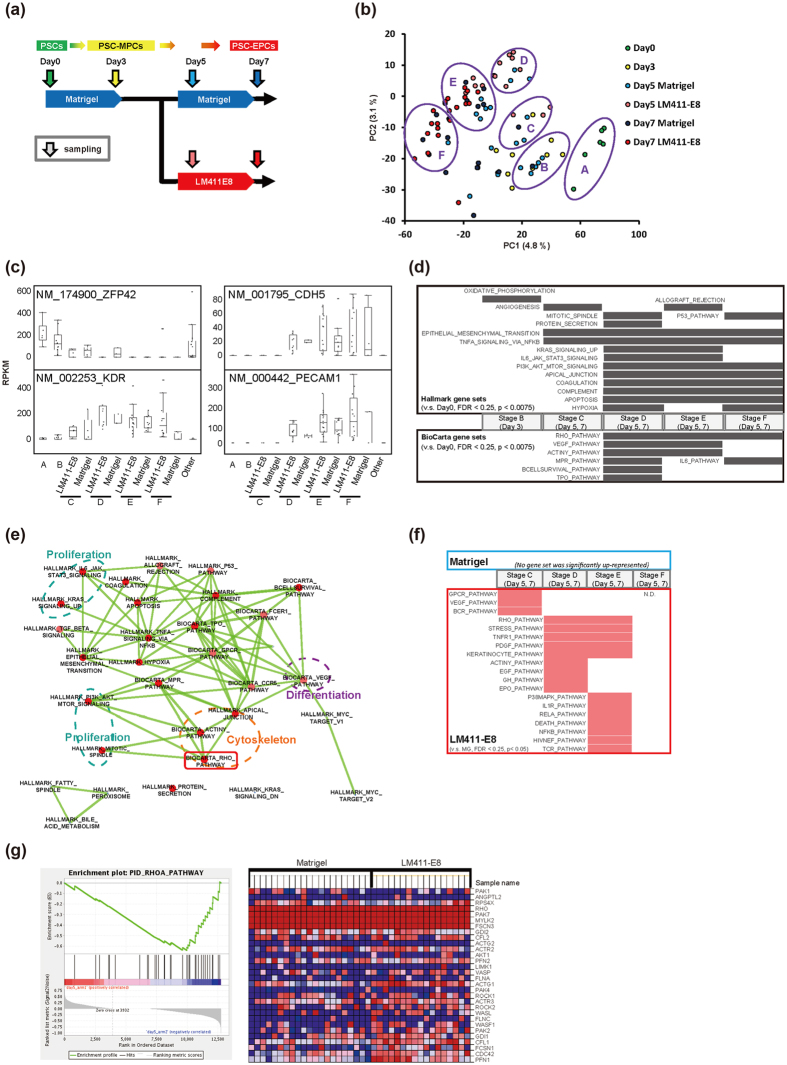
Single-cell RNA-seq analysis during endothelial differentiation. (**a**) Sampling time points for single-cell RNA-seq. (**b**) Cell heterogeneity in the process of differentiation on LM411-E8 or Matrigel evaluated using principal component analysis based on single-cell RNA-seq data (KhES-1). Each cell was classified into 6 stages (A–F). (**c**) Expression of undifferentiated cell marker ZFP42, mesodermal marker KDR, endothelial marker CDH5 and PECAM1 during differentiation. RPKM value of each gene was shown. (**d**) Gene set enrichment analysis (GSEA) following the orderly differentiation. Data sets of samples at stage B to F were independently compared to Stage A by GSEA. Genes with RPKM > 0 in at least one sample were applied to the analysis. Gene sets of significantly (FDR < 0.25, p < 0.0075) up-represented pathways at each stage in Hallmark (upper field) and BioCarta (lower field) were shown (size-filtered by 12–500). (**e**) An expanded enrichment map showing the relationship among gene sets up-represented at stage D. (**f**) Gene sets enriched in cells on Matrigel or LM411-E8. Data sets of samples at stage C to F were independently analyzed by GSEA. Genes with RPKM > 0 in at least one sample were applied to the analysis. Gene sets of significantly (FDR < 0.25, p < 0.05) up-represented pathways in BioCarta (size-filtered by 12–500) were shown. No pathway was significantly up-represented at stage F. (**g**) A representative result of GSEA of day 5 cells differentiated on Matrigel or LM411-E8 (KhES-1).

In order to investigate the effects of the matrices on endothelial differentiation at the single cell level, we next performed single-cell RNA-seq analysis. We plated PSC-MPCs onto fresh Matrigel- or LM411-E8-coated dishes and sampled single cells at different time points (Fig. 4a, Supplementary Fig. 8). Consequently, we detected a comparable number of genes in each cell (Supplementary Fig. 9a,b). Principal component analysis (PCA) depicted a seamless transition of the transcriptional profiles during differentiation at single-cell resolution (Fig. 4b). In this panel, we could putatively classify each sample into six differentiation stages: A (pluripotent stem cells), B (early mesodermal progenitors), C (mesoderm progenitors), D (early endothelial progenitors), E (intermediate endothelial progenitors), F (late endothelial progenitors) and others (Fig. 4b). According to this classification, expression of literature-based representative genes related to mesoderm and endothelial differentiation, such as KDR (NM_002253), CDH5 (NM_001795) and PECAM1 (NM_000442), were confirmed to be orderly up-regulated while a pluripotent stem cell marker ZFP42 (REX1, NM_174900) was down-regulated (Fig. 4c). Notably, the profiles of PSC-MPCs plated onto LM411-E8 were relatively homogenous and followed an aligned transition from day 5 to day 7. On the other hand, those on Matrigel were relatively heterogeneous, though a small subset of cells kept pace with those on LM411-E8 (Fig. 4b and Supplementary Fig. 10a). Overall, these results suggest that LM411-E8 facilitated uniform endothelial differentiation.

**Figure 5:**
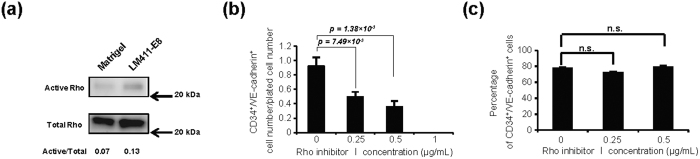
Activation of Rho pathway in PSC-EPCs on LM411-E8. (**a**) Evaluation of Rho activation using a pull-down assay in the lysates of day 5 cells differentiated on Matrigel (left lane) and LM411-E8 (right lane) (KhES-1). The chemiluminescence intensity ratios of active to total Rho were shown below gel images. (**b,c**) Cell number (**b**) and percentage (**c**) of PSC-EPCs treated with Rho inhibitor I during differentiation from day 3 (KhES-1). Data are presented as the mean ± SEM (n = 3) and were statistically analyzed using Student’s *t*-test. Representative results of at least two independent experiments are shown.

We next looked for the pathways contributing to endothelial differentiation in our culture. Gene set enrichment analysis (GSEA) on each stage compared to undifferentiated stage A revealed the highly significantly up-represented pathways (FDR < 0.25, p < 0.0075) along the orderly differentiation (Fig. 4d and Supplementary Table 1). As shown in Fig. 4d, GSEA indicated that our culture successfully transform initial differentiation cells (Stage B; oxidative phosphorylation was elevated from pluripotent stage, compatible to previous reports^20^,^21^) into endothelial stages (Stage C to F). Interestingly, expanded enrichment maps (Fig. 4e, Supplementary Fig. 10b,c) figured out the close relationships between cytoskeleton-related Rho pathway and proliferation-related pathways during differentiation, especially in earlier endothelial (D and E) stages. Moreover, additional GSEA analysis revealed that Rho pathway is significantly enriched in cells on LM411-E8 than those on Matrigel in stage D and E, suggesting the function of LM411-E8 in stimulating proliferation of early stage endothelial progenitors via Rho pathway (Fig. 4f,g, Supplementary Table 2). Indeed, though expression of Rho itself was not upregulated, its’ activated form in PSC-EPCs show the clear increase on LM411-E8 (Fig. 5a), and chemical inhibition of the Rho-GTPase pathway drastically decreased the yield of PSC-EPCs (Fig. 5b) while maintaining the high purity (Fig. 5c). Considering that Rho signaling pathway is activated downstream to VEGF as well as various adhesion molecules including integrins^22^,^23^, these data highlight the important role of the Rho signaling pathway in achieving a higher endothelial yield under LM411-E8 and VEGF co-existing conditions.

### Modification of initial mesodermal induction improved the efficiency of endothelial differentiation

**Figure 6:**
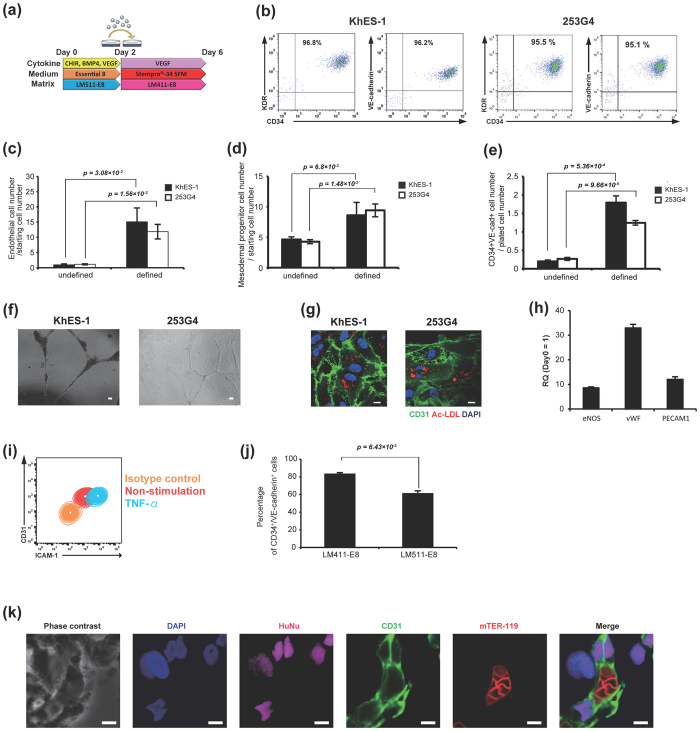
Improvement of differentiation efficiency. (**a**) Schematic process of the modified differentiation system via activation of the canonical Wnt signaling pathway. (**b**) Representative flow cytometry plots of the day 6 cell population (KhES-1). (**c**) Efficiency of endothelial differentiation in the modified system compared to the unmodified system. The ordinates indicate the number of PSC-EPCs per starting PSC number. Data are presented as the mean ± SEM (n = 3) and were statistically analyzed using Student’s *t*-test. Representative results of at least three independent experiments are shown. (**d**) The cell numbers on day 3 (undefined system) and day 2 (defined system), normalized by starting PSC numbers. The data were analyzed using Student’s *t*-test, n = 3. (**e**) CD34^+^VE-cadherin^+^ cell numbers at day 6. Cell numbers were normalized by plated cell numbers on LM411-E8. The data were analyzed using Student’s *t*-test, n = 3. (**f**) A tube formation assay of PSC-ECs. Scale bars: 200 μm. (**g**) Ac-LDL-uptake and CD31 expression of PSC-ECs. Scale bar: 10 μm. (**h**) Expression of vWF, eNOS and PECAM1 in PSC-EPCs compared to undifferentiated (day 0) cells. The RQ values were calculated via the ΔΔCt-method using GAPDH on day 0 as an internal control. (**i**) ICAM-1 expression in PSC-ECs stimulated with 10 ng/mL TNF-α for 18 hours. (**j**) The proportion of CD34^+^VE-Cadherin^+^ cells on LM411-E8 or LM511-E8. Day 2 cells were plated cultured for four days. The data were analyzed using Student’s *t*-test, n = 3. (**k**) Immunofluorescence images of Matrigel-plug sections. PSC-EPCs (KhES-1) generated according to the above method were suspended in Matrigel and subcutaneously transplanted to NOG mice. Human endothelial cells (positive for HuNu and CD31) can be seen surrounding murine erythrocytes (positive for TER-119). Scale bars: 5 μm.

Although our differentiation system produces PSC-EPCs with high purity, the yield of PSC-EPCs is not satisfactory (see Fig. 2c). We considered that this might be because the number of PSC-MPCs adapted to LM411-E8-guided differentiation system is still low. Therefore, to improve the yield of PSC-EPCs, we tried to modify the condition of initial differentiation into PSC-MPCs. Since endogenous activation of the Wnt-β-catenin pathway potentiates PSC differentiation towards the primitive streak and mesoderm^24^,^25^,^26^, we used CHIR99021, a potent inhibitor of GSK3β^27^, to activate the Wnt-β-catenin pathway during initial differentiation. Additionally, we adopted a perfectly chemically-defined medium and recombinant LM511-E8 coating during initial differentiation that is applicable to future regenerative medicine (Fig. 6a). This modified defined system maintained the same level of purity without cell sorting (Fig. 6b) but drastically increased the yield of PSC-EPCs, producing 10 to 15 PSC-EPCs from one PSC (Fig. 6c). The modified protocol increased the growth rate in initial differentiation (Fig. 6d). These findings were compatible with the results of a quantitative expression assay, in which the Wnt pathway genes leading to cell cycle activation were up-regulated in PSC-MPCs under defined condition compared to both undifferentiated PSCs and PSC-MPCs under original undefined condition (Supplementary Fig. 11a,b, Supplementary Tables 3 and 4). The expansion rate of PSC-EPCs from PSC-MPCs was also increased in the defined system (Fig. 6e). The tube formation assay and LDL-uptake assay verified that the PSC-ECs in this system were functional (Fig. 6f,g). Indeed, the representative genes deeply associated with the endothelial function, such as vWF, eNOS and PECAM1, were up-regulated in PSC-EPCs (Fig. 6h). Furthermore, the expression of ICAM-1, a leukocyte adhesion molecule, was up-regulated by TNF-α stimulation (Fig. 6i). Importantly, LM411-E8 still showed significantly higher purity of PSC-EPCs than LM511-E8 (Fig. 6j). This finding supported the importance of guiding function by LM411-E8 for mesodermal progenitor cells, even in the Wnt-activated condition. Finally, we confirmed that the PSC-ECs could give rise to functional blood vessels *in vivo*. In the Matrigel plug assay, the transplanted PSC-EPCs successfully presented with vascularization perfused with mouse blood, verifying the functional activity of PSC-EPCs plated on LM411-E8 *in vivo* (Fig. 6k).

## Discussion

Here we found that matrix switching onto LM411 and its C-terminal short fragments efficiently guides PSC-MPCs towards endothelial lineage. Our results highlight the importance of signal transduction through cell-matrix interaction for the fate decision of endothelial progenitor cells.

LM411-coating yielded PSC-EPCs at higher purity compared to Matrigel and the other laminin matrix LM511. The part of these results may be associated with the specific binding spectrum of LM411 to integrin family proteins. One possibility is that the narrower binding spectrum of LM411 may increase the apparent specification efficacy by excluding the deviant population at initial adhesion. As shown in the binding assay using specific blocking antibodies to integrin (Supplementary Fig. 6b), adhesion of PSC-MPCs onto LM411-E8 is exclusively dependent on integrin α6β1. Undesirable binding of LM511 to α3β1 and α7β1 in addition to α6β1^8^ may hold non-committed progenitors on the matrix. The other possibility is that integrin signaling other than α6β1 during differentiation hamper the commitment of PSC-MPCs into endothelial lineage. While integrin α6β1 facilitates endothelial commitment through activation of Etv2, integrin α3β1 inhibits this pathway^28^. Since both LM111, a major component of Matrigel, and LM511 bind integrin α3β1 in addition to α6β1, these matrices might transduce conflicting signals to the progenitor cells, thereby reducing their specific commitment. The results of double-switching assay (Fig. 3a,b) support this possibility, as PSC-MPCs selected on LM411 did not differentiate into endothelial lineage efficiently on subsequent culture on LM511 or Matrigel. RNA expression analysis provided us interesting suggestions about intracellular functions responsible for present results. Previous studies have pointed out Rho family proteins as an important mediator of endothelial cell functions such as angiogenesis and stabilization. Especially, recent studies showed that RhoA mediates VE-cadherin expression and localization, which in turn negatively regulate angiogenesis by blocking VEGF signal as homeostatic mechanism. Present study may suggest the specific role of LM411-integrin α6β1 axis for supporting continual signal transduction in specific situation, e.g. embryonic vasculogenesis. However, further investigation will be needed since the possibility that unknown off-target binding to LM411 required for efficient endothelial differentiation still remains.

The sequential switch from LM511-E8 to LM411-E8 in chemically defined medium successfully provided a novel defined system for inducing endothelial cells from PSCs, with more than 95% purity, without cell sorting: this rate is more efficient than that described in previous reports with a purity of 40%^12^, 55%^29^ and 55.5%^30^. The yield of PSC-EPCs per single PSC was approximately 10 to 15, which is also larger than most previous reports^31^. Although the yield in our system remains unsatisfactory, the possibility of combining our protocol with the scalable expansion of endothelial progenitor cells^11^ should be taken into account to facilitate future clinical applications.

In conclusion, the present study demonstrated the concept of matrix switching for the field of *in vitro* directed differentiation culture. In addition to the endothelial lineage, recent studies have reported LM511-E8^32^ and laminin 111^33^,^34^ to be useful matrix components for delivering midbrain dopaminergic neurons and hepatocytes, respectively. We therefore propose that sequential switching of defined ECMs depending on the lineage and stage of cells is a reasonable strategy for establishing efficient differentiation systems.

## Methods

### Study approval

All experimental protocols were carried out in accordance with the relevant guidelines and approved by the relevant committees. The use of human embryonic stem cells (ESCs) was approved by the Ministry of Education Culture, Sports, Science and Technology (MEXT) of Japan. The use of human iPSCs was approved by the Ethics Committee of Kyoto University, and informed consent was obtained in accordance with the Declaration of Helsinki. The study plan for recombinant DNA research was approved by the Recombinant DNA Experiments Safety Committee of Kyoto University. The experimental protocol was approved by the Animal Research Committee of the Center for iPS Cell Research and Application, Kyoto University. Animal care was provided by the Institute of Laboratory Animals of the Center for iPS Cell Research and Application, Kyoto University.

### Antibodies

Anti-human KDR antibodies (BioLegend), anti-human CD34 antibodies (Beckman coulter), anti-human VE-cadherin antibodies (eBioscience), sheep anti-human CD31 antibodies (R&D systems), FITC-conjugated anti-sheep IgG antibodies (Jackson ImmunoResearch), mouse anti-human nuclei antibodies (Millipore), Cy3-conjugated anti-mouse IgG antibodies (Jackson ImmunoResearch), Alexa-Fluor 647-labelled rat anti-mouse TER-119 antibodies (BioLegend), rat anti-human CD49f antibodies (GoH3, BD), anti-human CD29 antibodies (AIIB2; developed by Caroline H. Damsky and obtained from Developmental Studies Hybridoma Bank) were used in this study. Rabbit polyclonal antibody against ACID/BASE coiled-coil peptides was produced by immunizing with ACID/BASE coiled-coil peptides as an immunogen. HRP-conjugated streptavidin was purchased from Thermo Scientific (Rockford, IL).

### Reagents

The following reagents were obtained from the indicated manufacturers: bFGF (Wako), 4% paraformaldehyde (Wako), crystal violet (Wako), ES medium (ReproCELL), mTeSR1 (STEMCELL TECHNOLOGIES), trypsin (Life technologies), collagenase IV (Life technologies), KSR (Life technologies), TrypLE Express (Life technologies), Stempro34 SFM (Life technologies), DiI-Ac-LDL (Life technologies), Endothelial Serum-Free Medium (Life technologies), Laemmli buffer (Bio-Rad), LM411 (Biolamina), LM511 (Biolamina), Matrigel (BD), GFR-Matrigel (BD), Type IV collagen (BD), Cytofix (BD), Perm/Wash (BD), Fibronectin (Millipore), LM511-E8 fragment (Nippi), BMP4 (R&D systems), VEGF (R&D systems), RNeasy kit (QIAGEN), bovine serum albumin (BSA, sigma), DMEM/F12 (sigma), sodium dodecyl sulfate (SDS, Nacalai tesque) and CaCl_2_ (Nacalai tesque).

### Cell lines

The human ES cell line KhES-1 was kindly provided by Dr. Hirofumi Suemori (Institute for Frontier Medical Sciences, Kyoto University, Kyoto, Japan). The human iPS cell lines 253G4 and 409B2 were kindly provided by Dr. Shinya Yamanaka (Center for iPS Cell Research Application, Kyoto University, Kyoto, Japan). The control iPSC clone 223Q5 was named W1 in a previous report^35^.

### Maintenance of human pluripotent stem cells (PSCs)

PSCs were maintained on SNL feeder cells in ES medium supplemented with 5 ng/mL of bFGF. At passage, the cells were dispersed with CTK solution (0.25% trypsin, 0.1% collagenase IV, 20% KSR and 1 mM CaCl_2_) for 30 seconds at room temperature, and SNL feeder cells were removed as previously reported^36^.

### Dish and plate coating

BD falcon non-coating tissue culture plates and dishes were used in this study. The following matrices were diluted with phosphate-buffered saline (PBS) (−) and used for dish coating at the indicated concentrations unless otherwise described: LM411 (2 μg/cm^2^), LM511 (2 μg/cm^2^), GFR (growth factor reduced)-Matrigel (20 μg/cm^2^), fibronectin (2 μg/cm^2^), LM411-E8 (0.4 μg/cm^2^) and LM511-E8 (0.4 μg/cm^2^). Type IV collagen was diluted in 0.05 M HCl and used for coating at 10 μg/cm^2^. The dishes and plates were coated for 2 hours at room temperature and rinsed with PBS (−) before cell plating.

### Differentiation of PSC-EPCs

PSC clumps were plated onto GFR-Matrigel-coated plates at a density of 2 clumps/cm^2^ and cultured in mTeSR1. When the colonies grew to a diameter of 750 μm, the medium was replaced with fresh mTeSR1 containing BMP4 (80 ng/mL). After three days, the cells were dissociated to the single-cell level using TrypLE Express for 20 minutes at 37 °C and subsequently plated onto a matrix-coated plate and cultured for an additional four days in Stempro34SFM (Life technologies) containing VEGF (80 ng/mL).

In the modified system, PSC clumps were plated onto LM511-E8-coated plates at a density of 5 clumps/cm^2^ and cultured in mTeSR1. When individual colonies grew up to the diameter of 750 μm, the medium was replaced with Essential 8 (Life technologies) containing CHIR99021 (4 μM), BMP4 (80 ng/mL) and VEGF (80 ng/mL). After two days, the whole culture was dissociated, plated onto LM411-E8-coated plates and cultured for four days in Stempro34SFM containing VEGF (80 ng/mL).

### Flow cytometric analysis

Flow cytometric analysis data were collected using LSRFortessa (BD) and subsequently analyzed with the FlowJo software package (Treestar).

### LDL-uptake assay and immunocytochemistry

Cells were reacted with Ac-LDL in Endothelial Serum-Free Medium (ESFM) for 5 hours at 37 °C in 5% CO_2_ and then washed with PBS (−) twice, fixed with Cytofix for 5 minutes and permeabilized with Perm/Wash at room temperature for 30 minutes. The primary antibody reaction with sheep anti-human CD31 antibodies (1:10) was performed at 4 °C overnight, and the cells were then washed with Perm/Wash twice. The secondary antibody reaction with FITC-conjugated anti-sheep IgG antibodies (1:100) was performed at room temperature for one hour. After washing with Perm/Wash, fluorescence imaging was performed using a FluoView FV10i confocal microscope (OLYMPUS).

### Tube formation assay

The tube formation assay was performed as previously reported^28^. For gel preparation, 50 μL of Matrigel was dispensed into each well of a 96-well plate and incubated at 37 °C for 30 minutes. The cells were suspended in ESFM supplemented with 80 ng/mL of VEGF, seeded at a density of 4 × 10^4^/well and cultured overnight. For measurement of the tube length and number of blanching points, the Image J software package (NIH) was used.

### Matrigel plug assay

The Matrigel plug assay was performed according to a previous report with some modifications^37^. Briefly, 1 × 10^6^ cells suspended in 100 μL of GFR-Matrigel supplemented with bFGF (300 ng/mL) were subcutaneously injected into the back skin of immunodeficient NOG mice (approximately 6 weeks old)^38^. The Matrigel plugs were harvested 21 days after transplantation and subjected to immunofluorescent staining.

### Immunohistochemistry

Matrigel block was fixed with 4% paraformaldehyde overnight at 4 °C, equilibrated in 20% sucrose/PBS (−) overnight at 4 °C and frozen embedded into O.C.T. compound. The embedded block was sectioned at a thickness of 6 μm and adsorbed onto glass slides. The slides were dried in air, fixed with Cytofix for 5 minutes at room temperature and permeabilized with Perm/Wash for 30 minutes at room temperature.

The primary antibody reaction was carried out in Perm/Wash for 2 hours at room temperature using sheep anti-human CD31 antibodies (1:10), mouse anti-human nuclei antibodies (1:100) and Alexa-Fluor 647-labelled rat anti-mouse TER-119 antibodies (1:10). After washing with Perm/Wash twice, the secondary antibody reaction was carried out in Perm/Wash for 1 hour at room temperature using FITC-conjugated anti-sheep IgG antibodies (1:100) and Cy3-conjugated anti-mouse IgG antibodies (1:100). Images were obtained with a FluoView FV10i confocal microscope (OLYMPUS).

### RNA extraction and real-time quantitative RT-PCR analysis

RNA samples were first prepared by silica gel membrane-based spin-columns (RNeasy -Kit; Qiagen, Valencia, CA, USA) in accordance with the manufacturer’s instructions. RNAs were then subjected to an RT reaction followed by cDNA amplification in accordance with the previously reported method^39^ with minimal modification. For targeted detection of four genes (vWF, eNOS, PECAM1 and GAPDH) and pathway-focused profiling (PrimerArray® Wnt signaling pathway [#PH010] and PrimerArray® cell-cycle [#PH002], TaKaRa), quantitative RT-PCR experiments were performed with a StepOnePlus™ Real-Time PCR System (Applied Biosystems) using a SYBR Premix Ex Taq II kit (#RR820S, TaKaRa), in accordance with the manufacturer’s instructions. Quantitative assessment of the expression was performed using the standard ΔCT method applying GAPDH as a single internal control (targeted detection), or a combination of GUSB, HPRT1, PGK1, ACTB, GAPDH, TBP, B2M and PPIA for multiple references (PrimerArray).

The oligonucleotide primers for targeted detections were as follows: human vWF, 5′-gaa atg tgt cag gag cga tg-3′ and 5′-atc cag gag ctg tcc ctc a-3′; human eNOS, 5′-gct gaa gga tgg ctg gac-3′ and 5′-ttg gca tct tcg cat gtc-3′; human PECAM1, 5′-gca aca cag tcc aga tag tcg t-3′ and 5′-gac ctc aaa ctg ggc atc at-3′; human GAPDH, 5′-agc cac atc gct cag aca c-3′ and 5′-gcc caa tac gac caa atc c-3′.

### KEGG pathway mapping

Gene lists filtered by significant increases (p < 0.05, D00 vs. MPC) or two-fold differences (V2D03 vs. V3D02) were mapped to the human Wnt signaling pathway (KEGG hsa04310) in the KEGG database (http://www.genome.jp/kegg/).

### RNA sequencing

Total RNA was extracted using the RNeasy kit as described in previous subsection, and the quality was tested with Bioanalyzer (Agilent). Ribosomal RNA was depleted by RiboZero Gold (Epicentre). RNA libraries were prepared with the Illumina TruSeq Stranded Total RNA Sample Prep kit and sequenced in the 100 cycle Single-Read mode of HiSeq2500 (Illumina). All sequence reads were extracted in FASTQ format using BCL2FASTQ Conversion Software 1.8.4 in the CASAVA 1.8.2 pipeline and mapped to hg19 reference genes, downloaded on 10^th^ December, 2012, using TopHat v2.0.8b^40^ and quantified by RPKM for Genes^41^, downloaded on 19th October, 2012. Gene Ontology analysis was done using David v6.7^42^. Total RNA samples were applied to RNA sequencing, and the data were analyzed using the Gene Spring software package.

### Single-cell RNA sequencing

Cells were dispersed with TrypLE Express for 20 minutes at 37 °C. Single cells were sorted using FACS Aria II and dropped into 96-well plates filled with 10 μL of Reaction buffer of SMARTer Ultra Low Input RNA – HV kit (Clontech), followed by cDNA synthesis and amplification according to the manufacturer’s instruction. Sequencing libraries were constructed using Nextera XT DNA Sample Prep kit (Illumina). The libraries were sequenced and all sequence reads were extracted, mapped and quantified as above.

### Gene Set Enrichment Analysis (GSEA)

GSEA (http://software.broadinstitute.org/gsea/index.jsp) was used to estimate biological signatures. Data sets filtered by the criterion requiring RPKM > 0 in at least one sample were applied for each analysis with Molecular Signatures Database v5.1 (Hallmark and BioCarta gene sets, size-filters set at 12–500) using gene-set permutation. Networks were visualized by Cytoscape (http://www.cytoscape.org) (P-value Cutoff 0.05, FDR Q-value Cutoff 0.25 and Overlap Coefficient Cutoff 0.01 were used).

### GSE accession number

RNA-seq datasets deposited in the GEO database can be accessed with the GEO accession number GSE85784.

### Cell adhesion assay

The cell adhesion assay was performed as previously reported^10^. Ninety-six-well plates coated with matrices were blocked with 1% BSA in DMEM/F12 for 1 hour at room temperature. The cells were suspended in DMEM/F12 containing 0.1% BSA and plated onto coated wells. After 30 minutes of incubation, non-adherent cells were removed via pipetting with DMEM/F12 twice. The remaining adherent cells were fixed with 10% formaldehyde for 15 minutes at room temperature and post-fixed with 100% ethanol for 5 minutes at room temperature. The fixed cells were then stained with 0.4% crystal violet in methanol (100%) for 5 minutes at room temperature. After washing extensively with deionized water, the remaining crystal violet in the cells was eluted with 1% SDS and quantified by measuring the optical density at 570 nm using a multi-well plate reader. For the functional inhibition assay, cells were pre-incubated with neutralizing antibodies in DMEM/F12 containing 0.1% BSA for 30 minutes on ice before plating.

## Additional Information

**How to cite this article**: Ohta, R. *et al*. Laminin-guided highly efficient endothelial commitment from human pluripotent stem cells. *Sci. Rep*. **6**, 35680; doi: 10.1038/srep35680 (2016).

**Publisher’s note:** Springer Nature remains neutral with regard to jurisdictional claims in published maps and institutional affiliations.

## Supplementary Material

Supplementary Information

Supplementary Tables
