# Supplementary material for: Laminin-guided highly efficient endothelial commitment from human pluripotent stem cells
Source: Sci Rep. 2016 Nov 2;6:35680. doi: 10.1038/srep35680 (PMC5090224; doi:10.1038/srep35680)
Supplement: Supplementary Information [file srep35680-s1.pdf]

Supplementary Information

Laminin-guided highly efficient endothelial commitment from human pluripotent stem cells

*Ryo Ohta[1,5], Akira Niwa[1,5], Yukimasa Taniguchi[3], Naoya M. Suzuki[1], Junko Toga[3], Emiko Yagi[3], Norikazu Saiki[1], Yoko Nishinaka-Arai[1], Chihiro Okada[2,4], Akira Watanabe[2], Tatsutoshi Nakahata[1], Kiyotoshi Sekiguchi[3,**], and Megumu K. Saito[1,*]*

[1]Department of Clinical Application and [2]Depeartment of Life Science Frontiers, Center for iPS Cell Research and Application, Kyoto University, Kyoto, Japan
[3]Laboratory of Extracellular Matrix Biochemistry, Institute for Protein Research, Osaka University, Suita, Japan
[4]Mitsubishi Space Software CO., LTD, Amagasaki, Japan
[5]These authors contributed equally to this work
**,*Corresponding authors

Supplementary Fig. 1

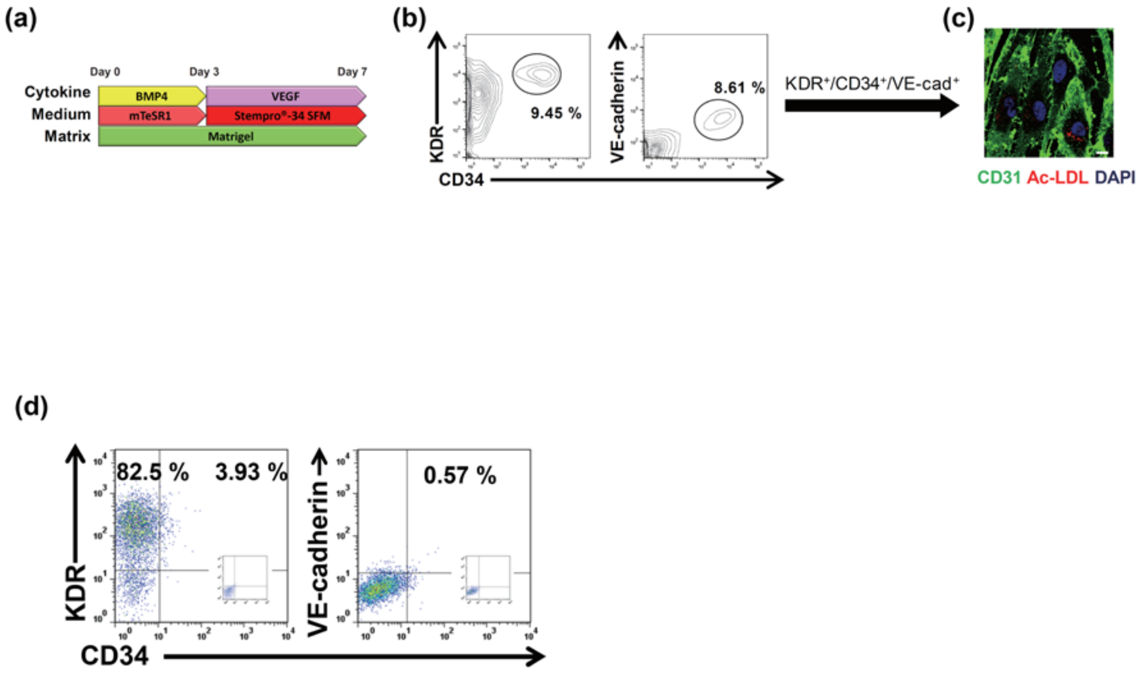

Supplementary Figure 1.
Endothelial cell induction in the conventional two-dimensional method.
(a) Schematic process of the conventional two-dimensional method for endothelial differentiation. (b) Representative flow cytometry plots of the day 10 cell population (KhES-1). (c) Representative image of Acetyl-LDL uptake by CD31-positive cells sorted using $KDR^{+}/CD34^{+}/VE\text{-}cadherin^{+}$(KhES-1). Scale bar: 10 µm. (d) Representative flow cytometry p
lots of day 3 cells.

Supplementary Fig. 2

(a)

KhES-1

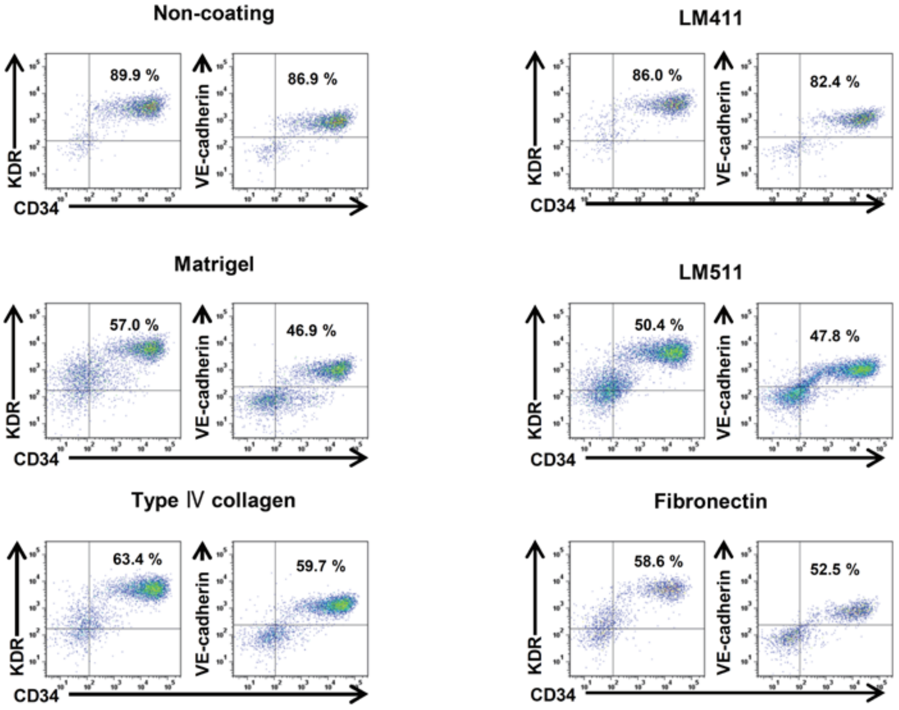

(b)

253G4

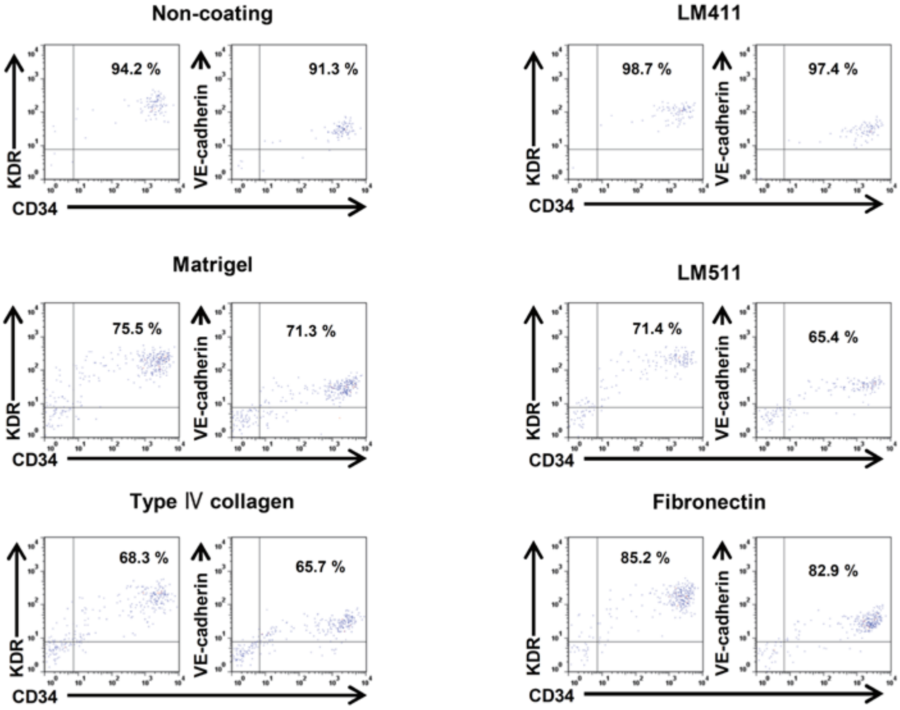

Supplementary Fig. 2 (continued)

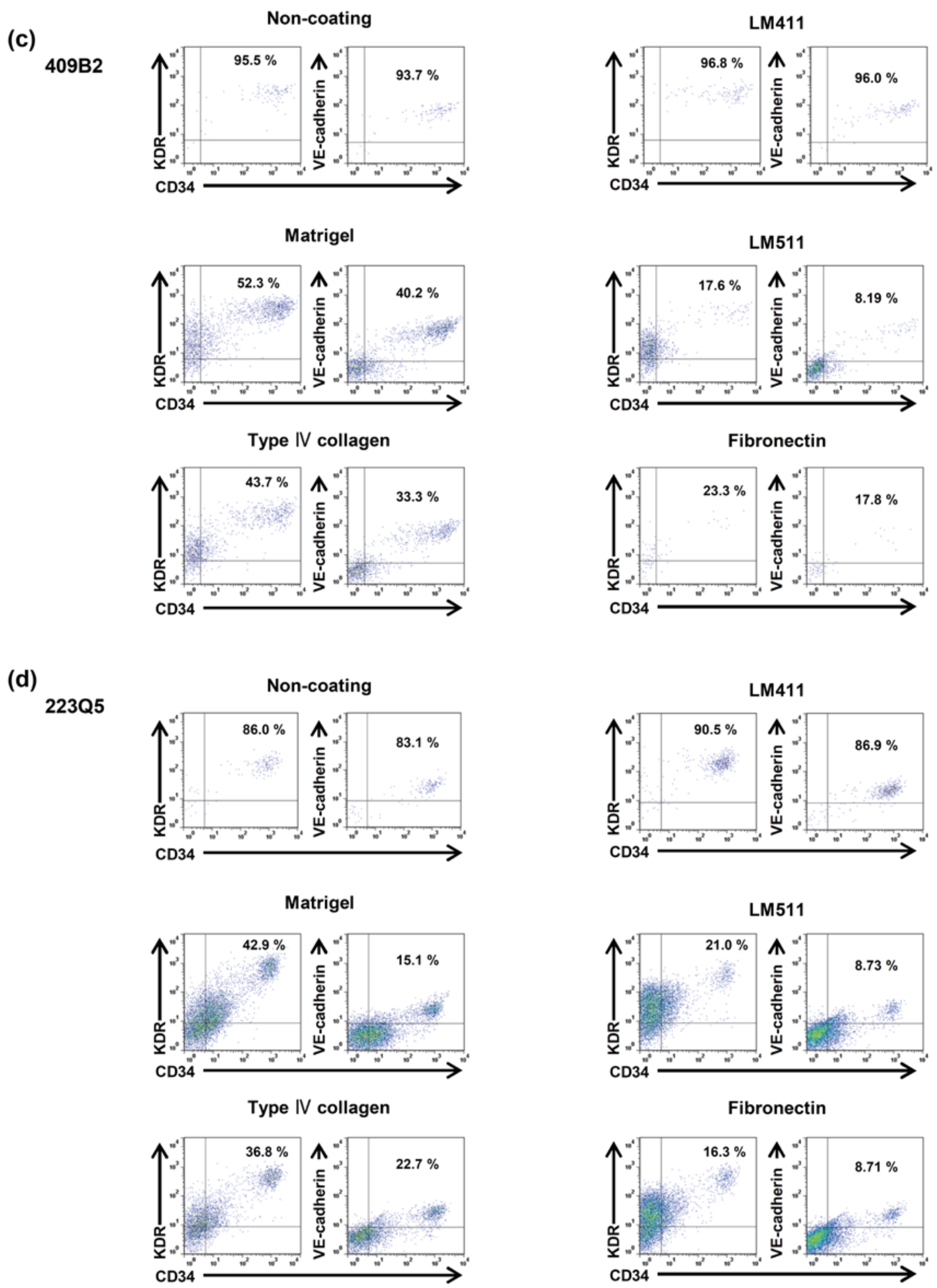

Supplementary Figure 2.

Purity of PSC-EPCs differentiated on various matrices.
Representative flow cytometry plots of day 7 cells differentiated on non-coated dishes , LM411, Matrigel, LM511, TypeⅣ collagen or fibronectin. (a) KhES-1. (b) 253G4. (c) 409B2. (d) 223Q5.

Supplementary Fig. 3

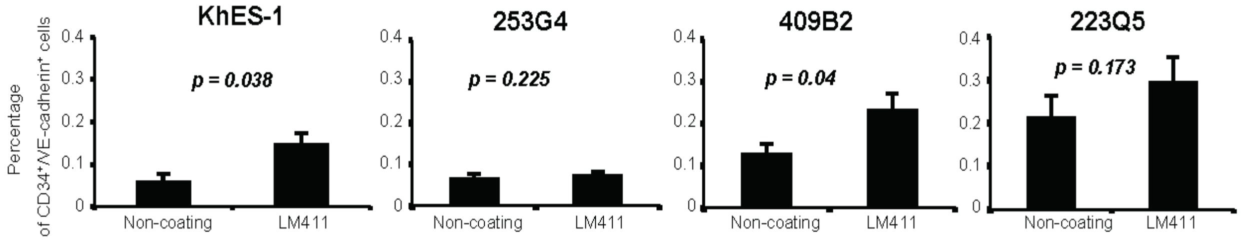

Supplementary Figure 3.
LM411 improved the yield of PSC-EPCs.
PSC-MPCs (day3) were plated onto non- or LM411-coated plates and differentiated for additional four days. The ordinates indicate the number of PSC-EPCs per day 3 cell number. Data are presented as the mean ±SEM (n=3) and were statistically analyzed using Student’s *t*-test.

Supplementary Fig. 4

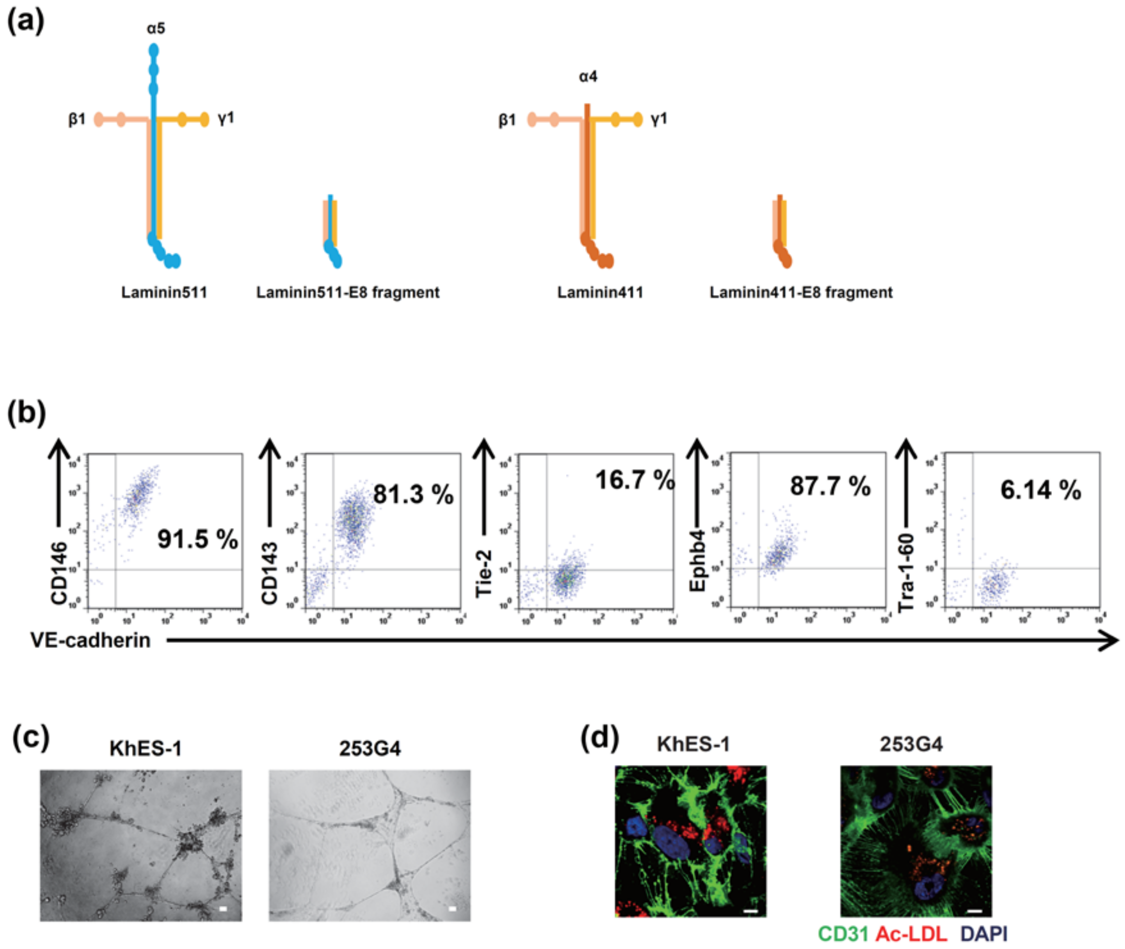

Supplementary Figure 4.
Phenotypes of PSC-EPC produced on LM411-E8.
(a) Schematic representations of two LM-E8s and the original intact laminin isoforms. (b) Expression of surface markers on PSC-ECs at day 9 (KhES-1) evaluated by flow cytometry. EC markers: CD146, CD143, Tie-2 and Ephb4, a PSC marker: Tra-1-60. (c,d) *In vitro* functionality of PSC-ECs produced on LM411-E8. (c) Images of tube formation of KhES-1 (left)- and 253G4 (right)- derived PSC-ECs. Scale bar: 200 μ m. (d) LDL-uptake and CD31 expression in the KhES-1 (left) - and 253G4 (right)-derived PSC-ECs. Scale bar: 10 µm.

Supplementary Fig. 5

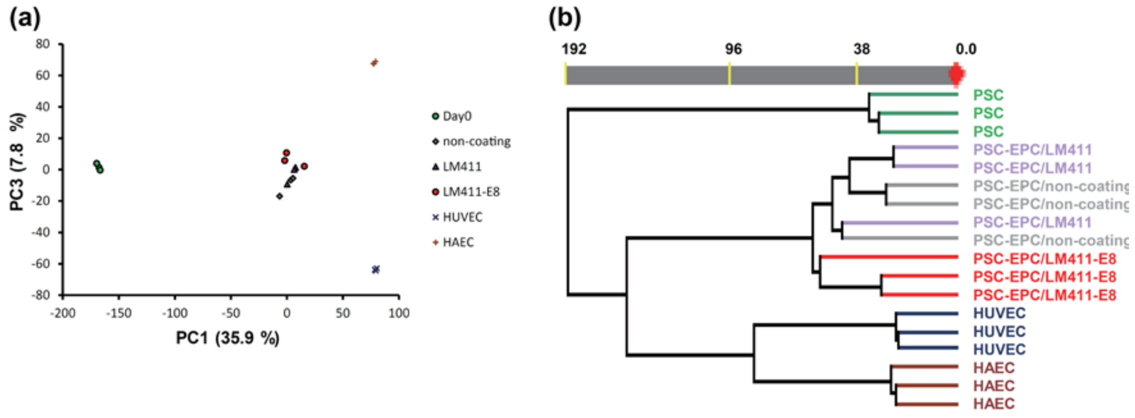

(c)

Term	PValue	Bonferroni	Benjamini	FDR
GO:0042310~vasoconstriction	7.21E-04	0.464182	0.464182	1.11239
GO:0035295~tube development	0.001712	0.772872	0.523421	2.622334
GO:0035239~tube morphogenesis	0.003285	0.941945	0.434064	4.9749
GO:0050880~regulation of blood vessel size	0.006929	0.997556	0.349225	10.22133
GO:0035150~regulation of tube size	0.006929	0.997556	0.349225	10.22133
GO:0003018~vascular process in circulatory system	0.008251	0.999228	0.361035	12.0568
GO:0001944~vasculature development	0.021062	1	0.48191	28.11512

Supplementary Fig. 5 (continued)

(d)

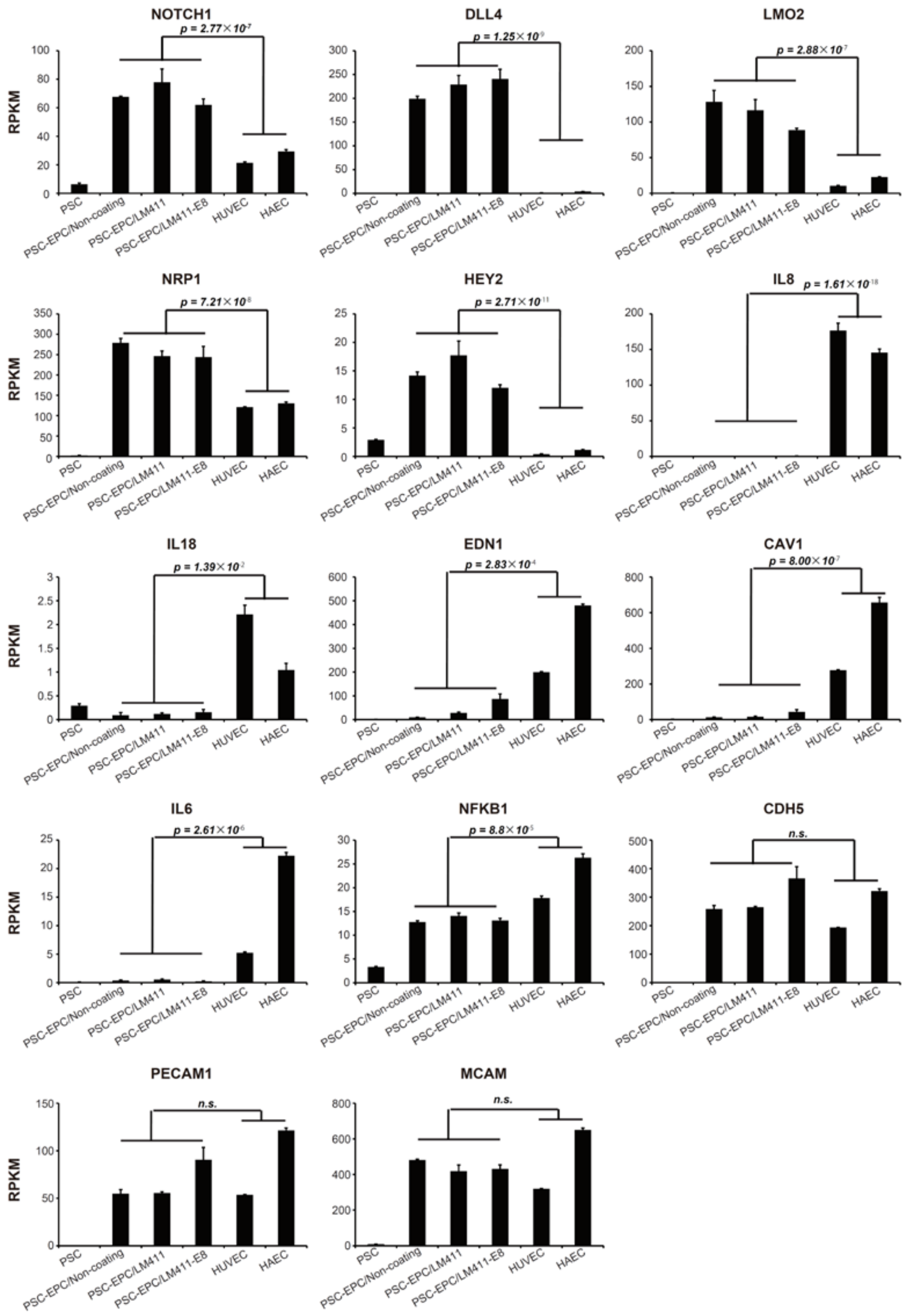

Supplementary Figure 5.
Gene expression profile of PSC-EPCs produced on LM411-E8 was closer to primary endothelial cells than the other PSC-EPCs.
(a) Gene expression profiles of KhES-1-derived PSC-EPCs differentiated on non-coated dishes, LM411 and LM411-E8 with undifferentiated KhES-1 cells and primary endothelial cells, HUVECs and HAECs were unbiasedly dissected using principal component analysis based on RNA-seq data. (b) Clustering of KhES-1 cells compared to PSC-EPCs differentiated from KhES-1 and the primary human endothelial cell lines HUVECs and HAECs (Euclidian distance, Average lincage). (c) Gene ontology analysis based on genes specifically increased by LM411-E8 versus the non-coating and LM411 groups. GO terms were extracted from DAVID’s library. (d) Expression of representative EC-associated genes. Expression (RPKM value) levels in PSC-EPCs and primary ECs are statistically analyzed by moderated t-test (P<0.05).

Supplementary Fig.6

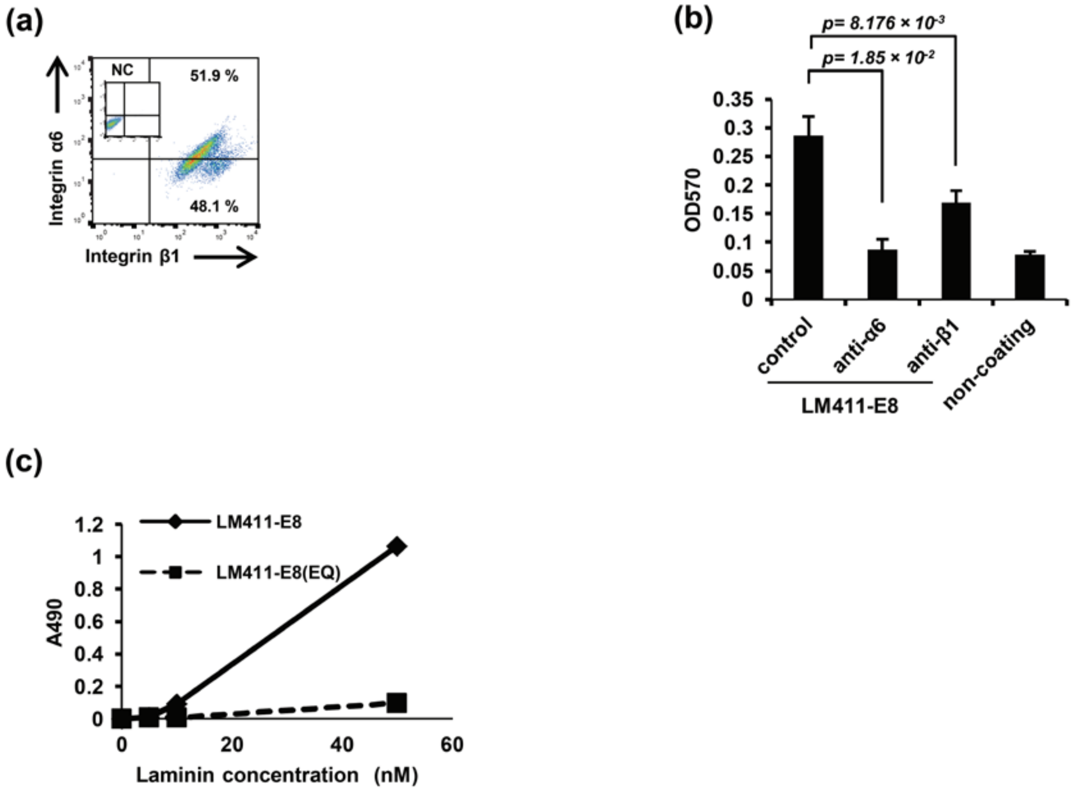

Supplementary Figure 6.
Receptor responsible for the initial adhesion to LM411-E8 and LM411-E8 (EQ).
(a) Representative flow cytometry plots of the day 3 cell population (KhES-1). The inset plot indicates negative control. (b) Inhibition of integrin α6 and β1 subunits on LM411-E8. Day 3 cells were incubated with integrin-specific neutralizing antibodies for 30 minutes on ice and seeded onto LM411-E8-coated plates. The concentrations of anti-integrin α6 and anti-integrin β1 antibodies were 2 μg/mL and 10 μg/mL, respectively. Data are presented as the mean ±SEM (n=3) and were statistically analyzed using Student's *t*-test. Representative results of at least three independent experiments are shown. (c) Loss of integrin α6β1 binding activity of LM411-E8(EQ). LM411-E8 dose-dependently increased the binding activity, whereas LM411-E8(EQ) did not. Data are presented as the mean ±SEM (n=3).

Supplementary Fig.7

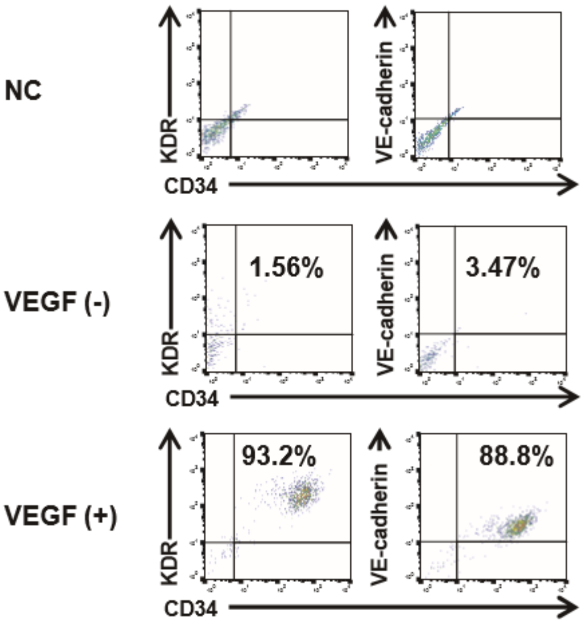

Supplementary Figure 7.
VEGF is required for endothelial differentiation on LM411-E8.
Representative flow cytometry plots of day 7 cells stimulated with or without VEGF (KhES-1). NC: negative control.

Supplementary Fig.8

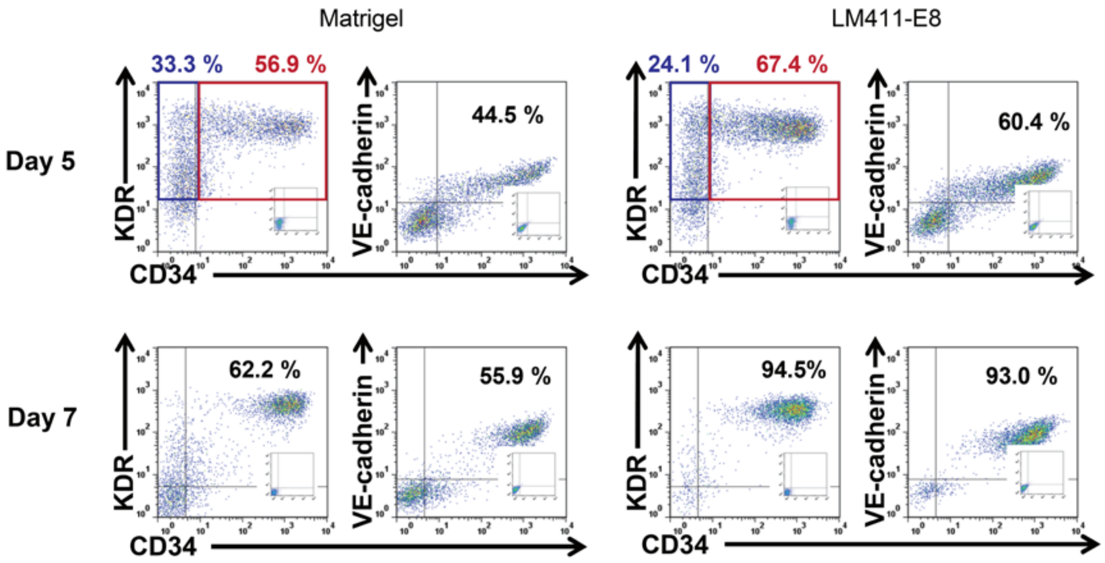

Supplementary Figure 8.
Endothelial marker expression in the process of differentiation.
Representative flow cytometry plots of days 5 and 7 cells. Day 3 cells were passaged onto Matrigel (left) or LM411-E8 (right) and cultured (KhES-1). Insets in each plot indicate negative controls.

Supplementary Fig.9

(a)

Sample Name	# of cells	# of Detected Genes (Mean±S.D.)	# of Mapped Reads
Day0	6	8625±1584	64,800,281±22,754,549
Day3	9	7419±745	63,949,717±25,826,000
Day5 Matrigel	21	7599±782	56,079,655±18,085,505
Day5 LM411-E8	17	8039±610	62,416,807±11,557,044
Day7 Matrigel	19	6430±1121	55,849,390±19,473,290
Day7 LM411-E8	20	5584±760	60,600,246±12,063,528

(b)

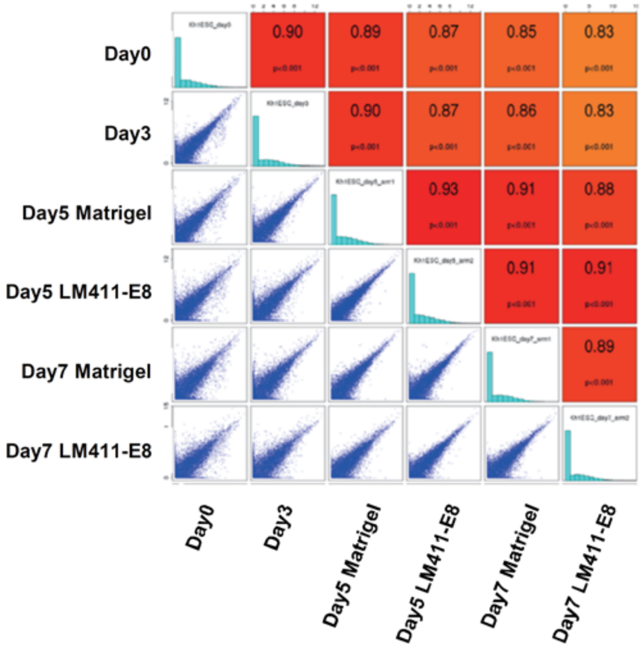

Supplementary Figure 9.
Basic characterization with single-cell RNA-seq.
(a) Number of cells and detected genes. (b) Pearson correlation matrix.

Supplementary Fig.10

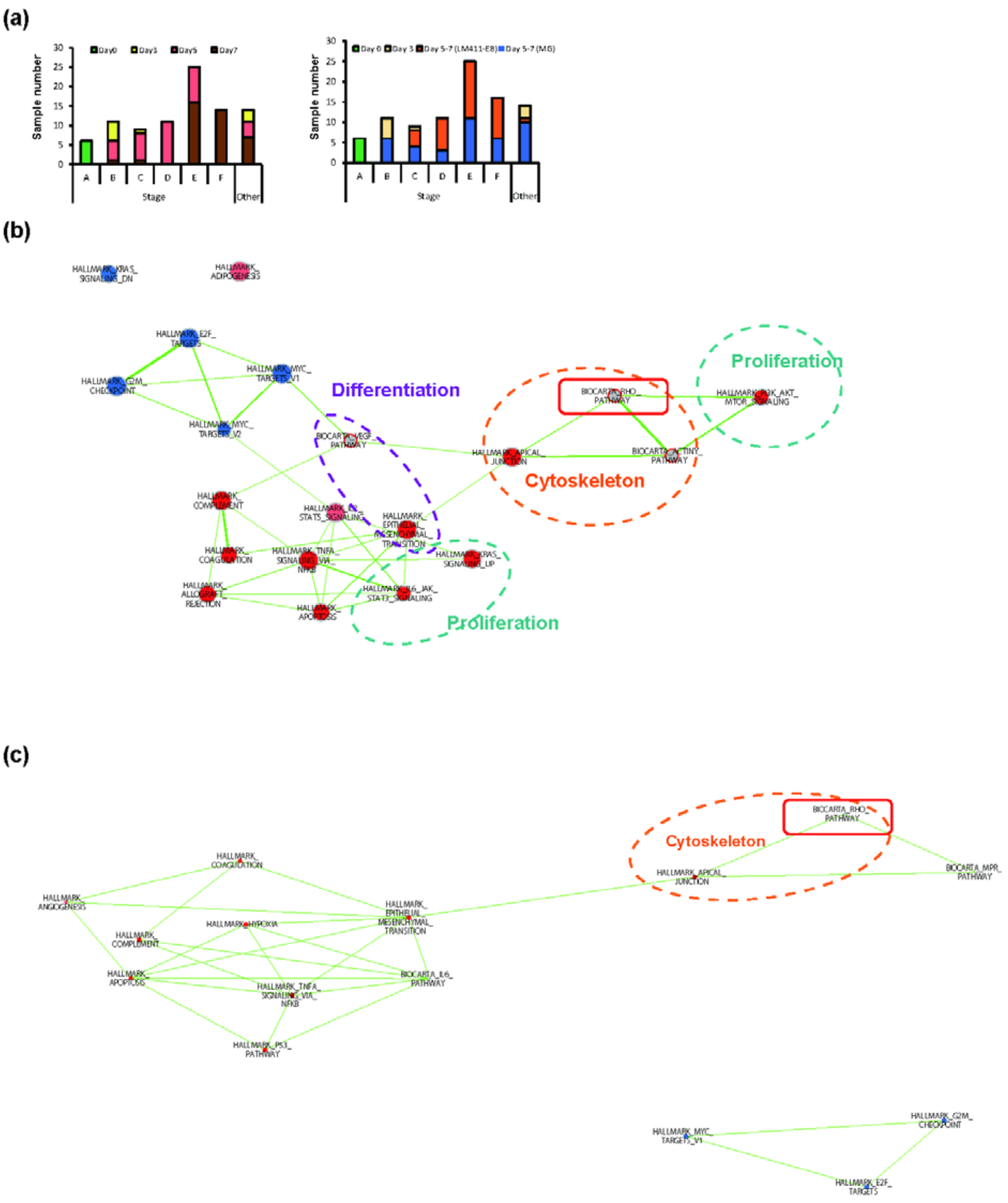

Supplementary Figure 10.
Additional data for single-cell RNA-seq.
(a) Sample distribution in each stage. Left panel shows distribution according to the time course. Right panel shows distribution according to the matrices used. (b,c) Expanded enrichment maps showing the relationship among gene sets up-represented at stage E (b) and F (c)

Supplementary Fig.11

(a)

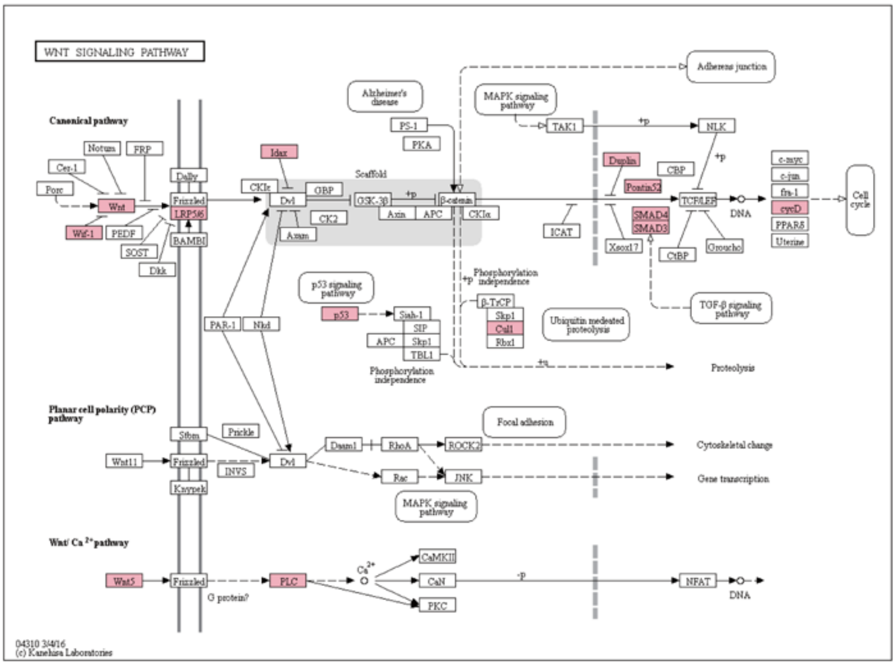

(b)

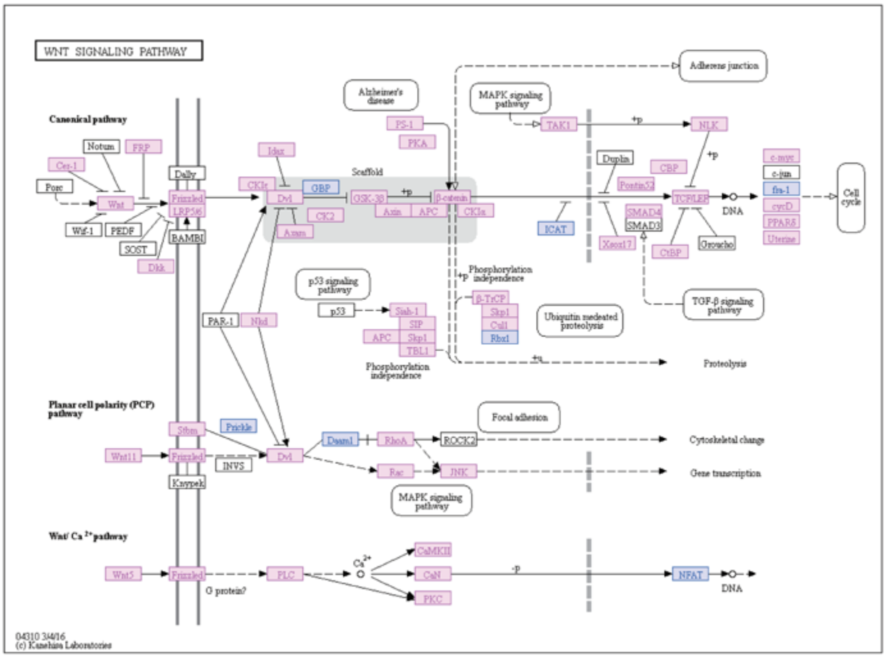

Supplementary Figure 11

Wnt pathway genes leading to cell cycle activation were more strongly up-regulated in PSC-MPCs produced under defined system

(a) Genes significantly upregulated ($p<0.05$, Welch's t test) in Wnt and cell-cycle pathway-focused panel during initial differentiation were mapped to KEGG database. (b) Genes selected by two fold up- and down- regulation in PSC-MPCs under defined system from those under undefined system were colored in magenta and blue, respectively.

Supplementary Methods

Rho activity assay

The Rho activity was measured with the Rho Activation Assay Kit according to the manufacturer's protocol. Briefly, 4.5×10^5 cells (day 5) were lysed in MLB and centrifuged at 10,000×g for 1 minute at 4 ℃. The supernatants were incubated with Rhotekin RBD for 1 hour at 4 ℃ with agitation. Then, the beads were washed with MLB and lysed in Laemmli buffer, and the protein lysates were subjected to SDS-PAGE.

SDS-PAGE and Western blotting

Equal numbers of cells were subjected to SDS-PAGE in Tris-Glycine buffer and transferred to PVDF membranes. The membrane was blocked with 2.5% skim milk in Tris-buffered saline containing 0.1% Tween-20 (TBS-T) for 1 hour at room temperature and probed with the appropriate primary antibody (3:1,000, mouse anti-Rho antibody, clone 55) for 2 hours at room temperature. After washing with TBS-T, the membrane was incubated with the appropriate secondary antibody (anti-mouse IgG HRP-linked antibody (1:1,000, cell signaling)) for 1 hour at room temperature. After washing with TBS-T, the membrane was incubated with West Femto super signal reagent (Thermo scientific), and the specific proteins were visualized with LAS-4000 (GE healthcare).

Recombinant LM411-E8 and its mutant fragment

A cDNA encoding murine Ig κ-chain leader sequence, 6XHis-tag and the E8 region of human α4 (Glu629-His1449 of NP_002281) was amplified by overlap extension PCR using a human laminin α4 expression vector [1] as a template for human α4 cDNA. The amplified DNA was treated with HindIII and EcoRI and ligated into the same restriction sites of pSecTag2B (Life Technologies, Carlsbad, CA).

Recombinant E8 fragments of laminin-α4β1γ1 and its γ1-Glu1607Gln mutant (designated as LM411-E8 and LM411-E8(EQ), respectively) were produced using FreeStyle™ 293 Expression System (Life Technologies). LM411-E8 and LM411-E8(EQ) were purified from conditioned media according to a method described previously[2]. Resultant proteins were dialyzed against phosphate-buffered saline without divalent cations, followed by sterilization through a 0.22 μm Millex-GV® filter unit (Millipore).

Solid-phase binding assay

Solid-phase binding assays of LM411-E8 and LM411-E8(EQ) were carried out using recombinant α6β1 integrin prepared according to a method described previously[3]. Briefly, a 96-well plate (Maxisorp, Nunc, Roskilde, Denmark) was coated with LM411-E8 or LM411-E8(EQ) at the indicated concentrations overnight at 4 °C, and blocked with TBS containing 1% BSA and 0.02% Tween-20 for 1 hour at room temperature. Following wash with TBS containing 0.1% BSA and 0.02% Tween-20 (wash solution), 30 nM of α6β1 integrin was added to each well and allowed to bind to adsorbed proteins for 3 hours at room temperature under 1 mM $MnCl_2$ or 1 mM $MnCl_2$/10 mM EDTA. Bound integrin was quantified by a colorimetric method after sequential incubations with the biotinylated anti-ACID/BASE antibody and HRP-conjugated streptavidin.

Statistical analysis

Statistical functions in Microsoft Excel 2010 were used for statistical analyses. Results are expressed as the mean ± SEM. Statistical significance was determined using Student's *t*-test, and P values <0.05 were considered significant.

Supplementary References

1. Hayashi, Y. et al. Identification and recombinant production of human laminin alpha4 subunit splice variants. *Biochem. Biophys. Res. Commun.* **299**, 498-504 (2002).
2. Ido, H. et al. The requirement of the glutamic acid residue at the third position from the carboxyl termini of the laminin gamma chains in integrin binding by laminins. *J. Biol. Chem.* **282**, 11144-11154 (2007).
3. Taniguchi, Y. et al. The C-terminal region of laminin beta chains modulates the integrin binding affinities of laminins. *J. Biol. Chem.* **284**, 7820-7831 (2009).
